# Design and development of a multi-functional bi-anisotropic metasurface with ultra-wide out of band transmission

**DOI:** 10.1038/s41598-021-03705-x

**Published:** 2021-12-20

**Authors:** Fahad Ahmed, Tania Tamoor, Tayyab Hassan, Nosherwan Shoaib, Akram Alomainy, Qammer H. Abbasi

**Affiliations:** 1 Research Institute for Microwave and Millimeter-Wave Studies (RIMMS), National University of Sciences and Technology (NUST), Islamabad, 44000 Pakistan; 2 School of Electronic Engineering and Computer Science, Queen Mary University of London, London, E1 4NS UK; 3 James Watt School of Engineering, University of Glasgow, Glasgow, G12 8QQ UK

**Keywords:** Engineering, Electrical and electronic engineering

## Abstract

This paper presents a multi-functional bi-anisotropic metasurface having ultra-wide out of band transmission characteristics. The proposed metasurface is comprised of 90° rotated T-shaped configuration yielding greater than or equal to 50% out-of-band transmission from above L- to X-band. Moreover, this metasurface achieves a maximum of 99% out-of-band transmission at lower frequency bands (i.e., L-band). The simultaneous absorptive and controlled reflection functionalities are achieved at 15.028 to 15.164 GHz along with polarization-insensitive and angular stable properties. The proposed metasurface yields state-of-the-art features compared to already published papers and has broader scope for Fabry Perot cavity, Radar cross-section (RCS) reduction, electromagnetic compatibility and interference (EMC/I) shielding, selective multi-frequency bolometers, ultrathin wave trapping filters, sensors and beam-splitters in the microwave domain.

## Introduction

In the last few years, the metasurfaces/frequency selective surfaces (FSSs) have spurred great intrigue among the research community owing to their unusual optical properties^1^. The metasurfaces are generally known as the two-dimensional analogs of bulky metamaterials^2^; where polarization, phase, and amplitude of incident light are manipulated via controlling the constitutive parameters. These extraordinary functionalities achieved in a miniaturized profile results in low system cost. Several compact metasurface-based devices such as filters^3^, polarizers^4,5^, and absorbers^6,7^ are realized in the literature. The absorptive anisotropic metasurfaces that are generally used to reduce radar cross-section (RCS) require a ground plane in their geometry. Although a reasonable absorption bandwidth and efficiency can be achieved through using such metallic ground planes; however, it creates two limitations:

1. Firstly, it inhibits the entire in-band and out of band transmission spectrum.
2. Secondly, metasurfaces become limited to only reflection and absorption-based functionalities (e.g. option for a transmission-based phenomenon to occur becomes impossible).

In view of the above, to avoid single functionality and hence zero transmission, reciprocal or non-reciprocal bi-anisotropic properties are essential in a structure. Such structures allow one to have different co-polarized reflections on both sides of the metasurface that further permits the realization of asymmetric absorption. One of the optimal bi-anisotropic metasurface that inherently meets such a performance requirement is known as an omega-type metasurface, and the property valid in this respect is known as omega coupling^8^. Generally, incorporating strong omega coupling in a reciprocal bi-anisotropic structure is quite difficult, however, modeling reasonably larger patch opposite to the absorptive side open the possibilities for asymmetric absorption (due to asymmetric reflections on both sides) along with non-zero in-band/out-of-band transmission^9,10^. A comparatively larger patch on backward illuminating side can resolve the out of band transmission issue, however, it limits the transmission power between 1 and 20%. Recently, an ultrathin bianisotropic metasurface is designed by creating hole in the structure, thus achieving 97% out of band transmission efficiency^11^. However, the achieved band is narrow, and the structure responds differently when exposed to circularly polarized wave. Therefore, the polarization insensitive bianisotropic omega metasurface with ultra-wide out of band transmission is still a topic of interest in scientific community.

In this paper, a cyclic-4 (90° rotated symmetry) T-shaped bi-anisotropic omega metasurface, having a hollow ring inside it, is presented. The same design is repeated on the opposing side of the absorptive side instead of larger metallic patches and creating holes. The proposed metasurface has the following functionalities:

1. Absorb the incident linear/circularly polarized (CP) wave in the frequency band of 15.028 to 15.164 GHz from one side along with ≤ 80% partial reflection on the other side.
2. Achieve ultra-wide out-of-band transmission in L-, S-, C- and X-band (to-date).
3. Attain maximum out-of-band transmission in L-band, which is almost 99% (salient feature).

The proposed metasurface also exhibits insensitive behavior toward any polarization and robust response against oblique incidences. The simultaneous absorption and controlled/partial reflection functionalities with high out-of-band transmission makes this design an appropriate applicant for not only Fabry Perot cavity/RCS reduction applications, but also for transmission-based applications, e.g., bandpass filters, beam splitters, and so forth.

## Metasurface design

### Geometrical configuration

It is evident from Ref.^12^, for perfect absorption, the sheet thickness should not be zero owning to the requirement of two sets of induced moments (i.e., magnetic moment and dipole electric) in the structure. A thin absorber can be designed with a set of linearly related dipole moment (magnetic ${m}_{y}$ and electric ${p}_{x}$) and incident field (magnetic ${H}_{iy}$ and electric ${E}_{ix}$) i.e.,

1 $${m}_{y}={\widehat{\alpha }}_{me}^{yx}{E}_{ix}+{\widehat{\alpha }}_{mm}^{yy}{H}_{iy },$$

2 $${p}_{x}={\widehat{\alpha }}_{ee}^{xx}{E}_{ix}+{\widehat{\alpha }}_{em}^{xy}{H}_{iy},$$

where, ${\widehat{\alpha }}_{em}^{cross}={\widehat{\alpha }}_{em}^{xy}, {\widehat{\alpha }}_{me}^{cross}={\widehat{\alpha }}_{em}^{xy}$, ${\widehat{\alpha }}_{mm}^{co}={\widehat{\alpha }}_{mm}^{yy}, {\widehat{\alpha }}_{ee}^{co}={\widehat{\alpha }}_{ee}^{xx}$ are effective cross electro-magnetic, cross magneto-electric, co-magnetic, and co-electric polarizabilities dyadic, respectively. To achieve the bianisotropic omega coupling in the structure, the metasurface should be asymmetric geometrically so that it can provide asymmetric response for reflections when excited from − z and + z directions (R^+z^ ≠ R^−z^)^9,10^. The thickness/dimension and geometry of the proposed metasurface are chosen in such a way that it does not violate the required conditions for both absorption phenomenon and bianisotropic omega functionality.

**Figure 1:**
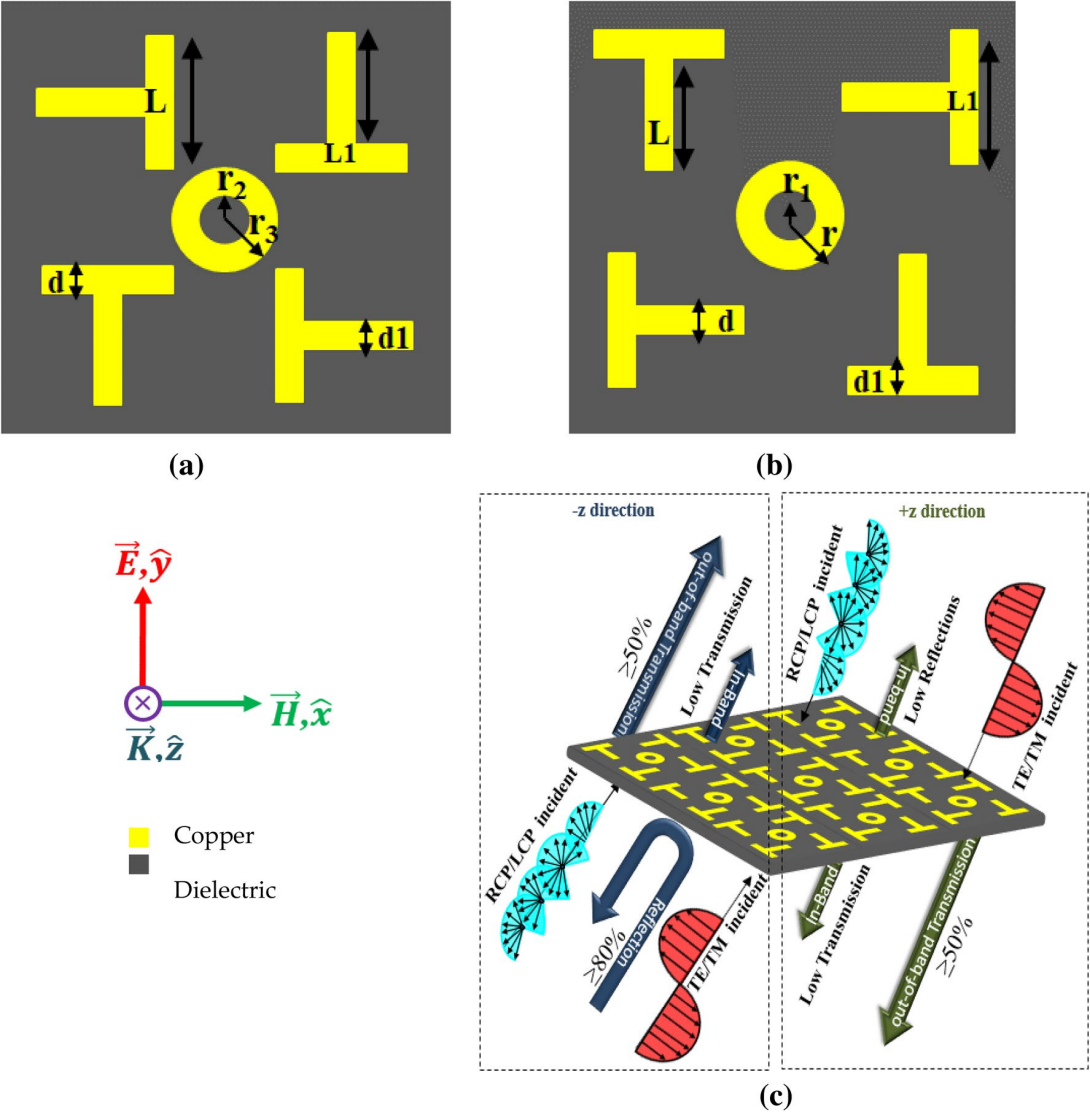
(**a**) Forward illuminating side. (**b**) Backward illuminating side. (**c**) Functionality depiction of proposed metasurface. (*The figure is created using Microsoft Power Point—Office 365—URL: https://www.office.com/powerpoint).

The proposed metasurface design is imprinted on both sides of the 2.4 mm FR-4 lossy substrate, as depicted in Fig. 1. The 90° rotated T-shaped four patches, and circular ring are placed on the upper side of the low-cost lossy substrate. The bottom side of the substrate is also printed by rotating the top side’s patches to 90° along their own axis and circular ring. The proposed unit cell is repeated along x- and y-direction with periodicity of p = 11.9 mm. The optimized dimensions of the proposed bianisotropic omega metasurface are L = 4 mm, L1 = 3.55 mm, d = 0.90 mm, d1 = 0.50 mm, r = r3 = 1.3 mm, and r1 = r2 = 1 mm.

### Theoretical background

A typical absorber requires the existence of a metallic ground plane; therefore; it is one-sided with zero out of band transmission^13^. Conversely, the absence of a ground plane allows having:

1. Both sided absorption along with non-zero out of band transmission.
2. One-sided absorption/controlled reflection along with non-zero out of band transmission.

To achieve both sided absorption, all the polarizability dyadic (electromagnetic/magnetoelectric coefficients) must be zero (${\widehat{\alpha }}_{em}^{cross}= {\widehat{\alpha }}_{me}^{cross}$ $={\widehat{\alpha }}_{em}^{co}= {\widehat{\alpha }}_{me}^{co}$ = 0) whereas co-electric and magnetic polarizabilities (${\widehat{\alpha }}_{ee}^{co}$ and ${\widehat{\alpha }}_{mm}^{co}$) should be balanced as Huygens’ pair, i.e.,

3 $${\widehat{\alpha }}_{ee}^{co}=\frac{S}{j\omega {\eta }_{0}}, {\widehat{\alpha }}_{mm}^{co}={{\eta }_{0}}^{2} {\widehat{\alpha }}_{ee}^{co}.$$

The effective co-electromagnetic, and co-magnetoelectric polariziabilities dyadic are represented by ${\widehat{\alpha }}_{em}^{co},\text{ and }{\widehat{\alpha }}_{me}^{co}$. The unit cell area, wave impedance, surface current and frequency can be denoted as S, ${\eta }_{0}\left(\sqrt{\frac{\mu }{\epsilon }}\right)$, $j$ and $\omega$ respectively. The absence of cross-coupling polarizabilities, in both sided absorbent structure, limit the functionality merely to absorption. The bianisotropy term infers as bi-polarization, and anisotropic behavior of a surface due to the existence of magnetoelectric ${(\widehat{\alpha }}_{me}={\widehat{\alpha }}_{me}^{cross}+{\widehat{\alpha }}_{me}^{co} )$ and electromagnetic coupling $({\widehat{\alpha }}_{em}= {\widehat{\alpha }}_{em}^{cross}+{\widehat{\alpha }}_{em}^{co})$ or cross-coupling polarizabilities. It not only provides a matchless opportunity for attaining single-sided absorption, but additionally provides an extra control on reflection/transmission on the opposing side along with out of band transmission.

The single-sided absorbers can be modeled through both bi-anisotropic-reciprocal and nonreciprocal structures. In reciprocal chiral and omega structures, the cross magnetic and electric polarizability dyadic must be zero (${\widehat{\alpha }}_{mm}^{cross}$ = 0, ${\widehat{\alpha }}_{ee}^{cross}=0$) and coupling (field) coefficients must satisfy ${\widehat{\alpha }}_{em}^{co} = -\,{\widehat{\alpha }}_{me}^{co}$ and ${\widehat{\alpha }}_{em}^{cross} = {\widehat{\alpha }}_{me}^{cross}$ conditions, respectively. Similarly, in non-reciprocal Tellegen and moving particle structures, the field coupling must correspondingly satisfy ${\widehat{\alpha }}_{em}^{co}={\widehat{\alpha }}_{me}^{co}$ and ${\widehat{\alpha }}_{em}^{cross}=-\,{\widehat{\alpha }}_{me}^{cross}$ conditions. To avoid external biasing and active components in the structure (i.e., increase fabrication complexities and cost), one can achieve such functionalities via reciprocal structure.

The incident EM wave behavior on these four types of bianisotropic metasurface is presented in Ref.^14^. The chiral property (${\widehat{\alpha }}_{em}^{co}=-\,{\widehat{\alpha }}_{me}^{co}$) provides reciprocal control on cross reflections (Rcr) and transmissions (Tcr). The omega functionality (${\widehat{\alpha }}_{em}^{cross}$ = ${\widehat{\alpha }}_{me}^{cross}$) offers control on co-reflection (i.e. R + z ≠ R-z, T + z = T-z). Similarly, Tellegen property (${\widehat{\alpha }}_{em}^{co}={\widehat{\alpha }}_{me}^{co}$) gives non-reciprocal control on cross reflections (Rcr) and transmissions (Tcr). The artificial moving functionality (${\widehat{\alpha }}_{em}^{cross}=-\,{\widehat{\alpha }}_{me}^{cross}$) provides control on co transmission (i.e., T + z ≠ T-z, R + z = R-z).

**Figure 2:**
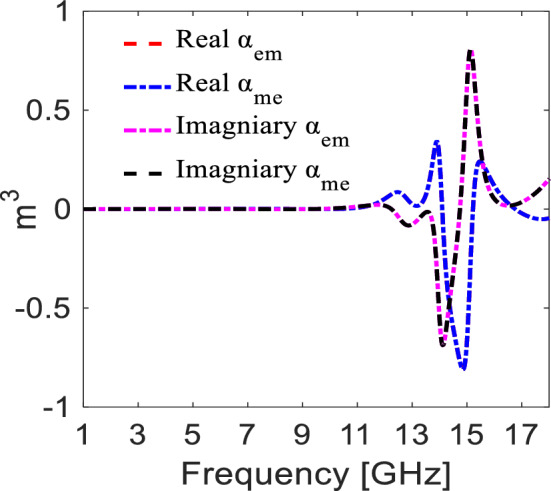
Depicting the real and imaginary part of cross-coupling polarizabilities (*The figure is created using MATLAB ver. R2020A—URL: https://www.mathworks.com/products/matlab.html).

To simultaneously design a one-sided absorber (forward side) and controlled/partial reflector (backward side) along with non-zero out-of-band transmission via bi-layered structure requires bi-anisotropic omega coupling (i.e. ${\widehat{\alpha }}_{em}^{cross}$ = ${\widehat{\alpha }}_{me}^{cross}$). We have retrieved and plotted the polarizability graphs to validate the presence of omega cross-coupling in the structure^15^. It can be seen from Fig. 2 that the conditions required for omega cross-coupling polarizabilities are fulfilled at 15.1 GHz.

### Metasurface performance

#### Reflection/transmission coefficients

**Figure 3:**
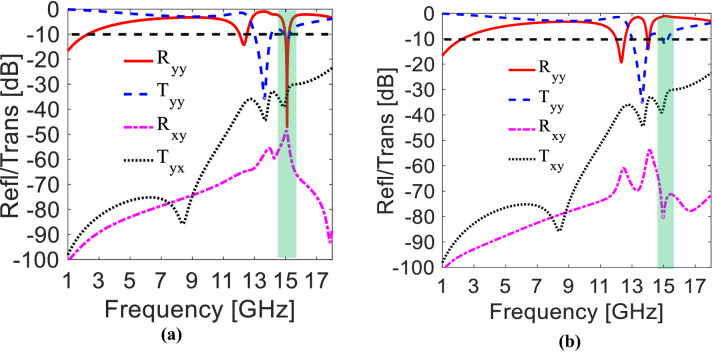
(**a**) Absorptive side. (**b**) Partially reflective side (*The figure is created using MATLAB ver. R2020A—URL: https://www.mathworks.com/products/matlab.html).

The proposed T-shaped metasurface performance is analyzed by performing a full-wave simulation on electromagnetic design tool CST MW Studio^®^ using periodic boundary conditions. The co- (i.e., ${{\varvec{R}}}_{{\varvec{y}}{\varvec{y}}}=|{{\varvec{E}}}_{{\varvec{r}}{\varvec{y}}}|/|{{\varvec{E}}}_{{\varvec{i}}{\varvec{y}}}|$ and ${{\varvec{T}}}_{{\varvec{y}}{\varvec{y}}}=|{{\varvec{E}}}_{{\varvec{t}}{\varvec{y}}}|/|{{\varvec{E}}}_{{\varvec{i}}{\varvec{y}}}|$) and cross-components (i.e., ${{\varvec{R}}}_{{\varvec{x}}{\varvec{y}}}=|{{\varvec{E}}}_{{\varvec{r}}{\varvec{x}}}|/|{{\varvec{E}}}_{{\varvec{i}}{\varvec{y}}}$| and ${{\varvec{T}}}_{{\varvec{x}}{\varvec{y}}}=|{{\varvec{E}}}_{{\varvec{t}}{\varvec{x}}}|/|{{\varvec{E}}}_{{\varvec{i}}{\varvec{y}}}$|) of reflected and transmitted fields are demonstrated in Fig. 3a, respectively. For complete absorption, the acceptance criterion for reflected and transmitted fields is taken − 10 dB. When y-polarized E-field is incident on the forwarding side, it is observed that all the transmitted and reflected fields remained lower than − 10 dB between 15.028 to 15.164 GHz as depicted in Fig. 3a. The controlled reflection from the opposite illuminating side is seen nearer to 80% at 15.1 GHz and rest of the bands are ≤ 50%, demonstrated in Fig. 3b. Moreover, the out-of-band transmission acquiring no less than 50% (− 3 dB) power in L- to X-band. It is important to discuss here that the maximum out-of-band transmission power (~ 0 dB) is attained at L-band.

#### Equivalent model

**Figure 4:**
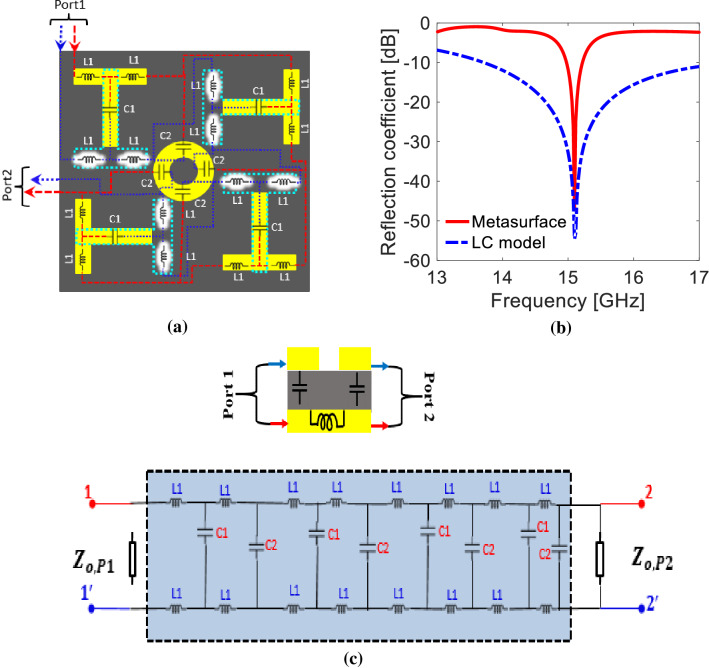
(**a**) Impedance model. (**b**) Resonance comparison of metasurface and LC model. (**c**) Equivalent circuit (*The figures (**a,c**) are create using Microsoft Power Point—Office 365—URL: https://www.office.com/powerpoint; while the figure (**b**) is created using MATLAB ver. R2020A—URL: https://www.mathworks.com/products/matlab.html).

The LC model is designed to observe the resonance of the proposed metasurface. To examine the analogous of unit cell to transmission line, two port (port 1 and port 2) are positioned on the LC circuit model as demonstrated in Fig. 4a. The end nodes of both ports are connected to upper and lower portion of the unit cell. The signal entering from one port and leaving from other port facing the equal impedance as experienced by the complete metasurface. The capacitance (i.e. the conductive lengths which have an equal metallic area on the opposite side are modeled as a capacitor) and inductance (i.e. the conductive lengths, where no conductor is present on the opposite side, are modeled as an inductor) in the structure starting from one port to another port are shown in Fig. 4a. The LC model and equivalent circuit is designed and simulated on ADS (Advanced Design System) as shown in Fig. 4b,c to attain the resonance frequency. The values of inductor (L1 = 0.318 nH) and capacitors (C1 = 0.1306 pF and C2 = 0.07 pF) are calculated using flat wire inductance and parallel plate capacitance equations (see Eqs. (4), (5)), respectively^16^. The resonant frequency (i.e. ${\text{f}}_{\text{r}}=1/2\uppi \sqrt{\text{LC}}$) analysis provides an additional benefit to strengthen the bianisotropy, reflection/transmission control, and frequency tuning by varying capacitance and inductance present in the structure.

4 $$\text{C} = \text{A}\upvarepsilon/\text{d},$$

5 $${L}_{flat}=2 \times {10}^{-4}l\left[\text{ln}\left(\frac{2l}{w+t}\right)+0.5+0.2235\left(\frac{w+t}{l}\right)\right],$$

where *L*_*flat*_: inductance of the conductor (μH), *l*: length of the conductor (mm), *w*: width of the conductor (mm), *t*: thickness of the condctor (mm).

#### Absorption

**Figure 5:**
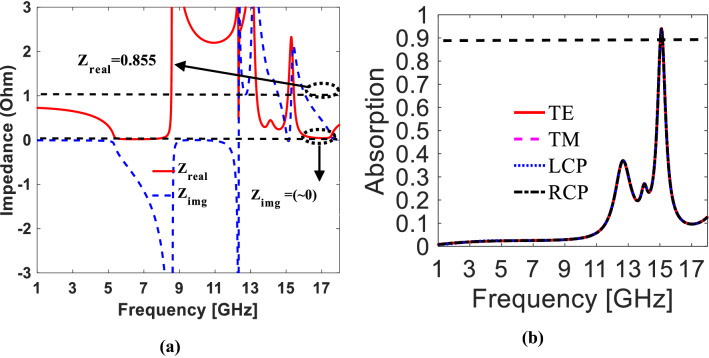
(**a**) Effective impedance of proposed metasurface. (**b**) Linear and Circularly polarized incident waves (*The figure is created using MATLAB ver. R2020A—URL: https://www.mathworks.com/products/matlab.html).

The absorption of an absorber can be characterized using the following equations:

6 $$\text{A }\left(\upomega \right)=1-\text{R}\left(\upomega \right)-\text{T}\left(\upomega \right),$$

7 $$\text{A }\left(\upomega \right)=1-{\left|{\text{S}}_{11}\left(\upomega \right)\right|}^{2}-{\left|{\text{S}}_{21}\left(\upomega \right)\right|}^{2},$$

where $\text{A}\left(\upomega \right),$ $\text{R}\left(\upomega \right),$ and $\text{T}\left(\upomega \right)$ are absorption, reflection, and transmission, respectively^17^. For perfect absorption, both the transmitted and reflected components should be zero. However, transmission is controlled through 90° rotated T-shaped proposed bi-anisotropic surface (i.e., no larger patch used). The major issue is to minimize reflections through the impedance matching technique. In this context, the Fresnel formula of reflectivity is given below:

8 $${{\left|{S}_{11}\right|}^{2}=\left|\frac{Z\left(\omega \right)-{Z}_{0}\left(\omega \right)}{Z\left(\omega \right)+{Z}_{0}\left(\omega \right)}\right|}^{2},$$

where, ${Z}_{o}$ is the characteristic impedance of free space which is equal to 377 Ω and $Z\left(\omega \right)$ is the impedance of metasurface. According to (8), maximum absorption can be achieved if $Z\left(\omega \right)$ approaches nearly equal to ${Z}_{0}\left(\omega \right)$. We have calculated the impedance (Z) of forwarding illuminating side of the proposed metasurface. It can be seen from Fig. 5a, the real part of the impedance is ${Z}_{real}=0.855\times 377=318.5$ Ω, while the imaginary part is ${Z}_{img}\sim 0$ at 15.1 GHz. By substituting the values in (8), we obtained ${\left|{S}_{11}\right|}^{2}$ = (0.08)^2^. The calculated value of ${\left|{S}_{11}\right|}^{2}$ = (0.08)^2^ and extracted value of ${\left|{S}_{21}\right|}^{2}$= (0.015)^2^ are further substituted in (7), which results in adsorption nearer to 95%.

To verify the absorption for $y$-polarized (TE), and right-handed circularly polarized (RCP) incident waves, the absorption graphs are plotted by modifying (7) to $A\left(\omega \right)=1-{\left|{R}_{yy}\right|}^{2}-{\left|{R}_{xy}\right|}^{2}-{\left|{T}_{yy}\right|}^{2}-{\left|{T}_{xy}\right|}^{2}$ and $A\left(\omega \right)=1-{\left|{R}_{++}\right|}^{2}-{\left|{R}_{-+}\right|}^{2}-{\left|{T}_{++}\right|}^{2}-{\left|{T}_{-+}\right|}^{2}$ respectively. The subscripts “ + ” and “−” represents right-handed and left-handed circularly polarized waves. The same is the case with $x$-polarized (TM) and left-handed circularly polarized (LCP) impinging waves. The absorption for all the polarization is demonstrated in Fig. 5b. The calculations and plotted graphs depicting almost similar values for absorption i.e., 95%.

#### Polarization insensitivity and angular stability

**Figure 6:**
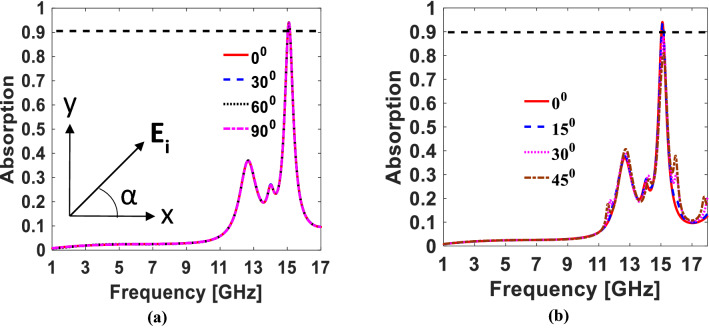
Absorption for (**a**) Polarization between TE and TM. (**b**) Oblique incidences (*The figure is created using MATLAB ver. R2020A—URL: https://www.mathworks.com/products/matlab.html).

Many potential applications require polarization insensitivity and angular stability against the electric field and incident angle rotation^18,19^. The polarization independence is mainly accredited to the symmetry existing in the unit cell configuration. A twofold (cyclic-2) and symmetry breaking configuration in the structure limits the metasurface response only to specific polarization. On the other hand, a cyclic-4 (i.e., 90° rotated) symmetric structure gives the same response against any electric field variation^10,20^. The proposed T-shaped configured structure (cyclic-4) qualifying the polarization insensitivity test when excited by an arbitrary linearly polarized impinging wave, as represented in Fig. 6a.

The same response of any metasurface against oblique incidence depends upon the following three factors:

1. Unique unit cell geometry.
2. Sub-wavelength unit cell size.
3. Low dielectric thickness.

The low dielectric thickness and small electrical length are weaker participants of angular stability. The factor that mainly plays a crucial role in angular stability is the unit cell’s unique geometry. To achieve possible stability, the thickness of the dielectric substrate and the unit cell size is kept smaller than the operating wavelength. Furthermore, the angular stability of a metasurface is improved by designing a unique T-shaped geometrical configuration. The proposed design is angularly stable up to 45° as depicted in Fig. 6b.

## Fabrication and measurements

**Figure 7:**
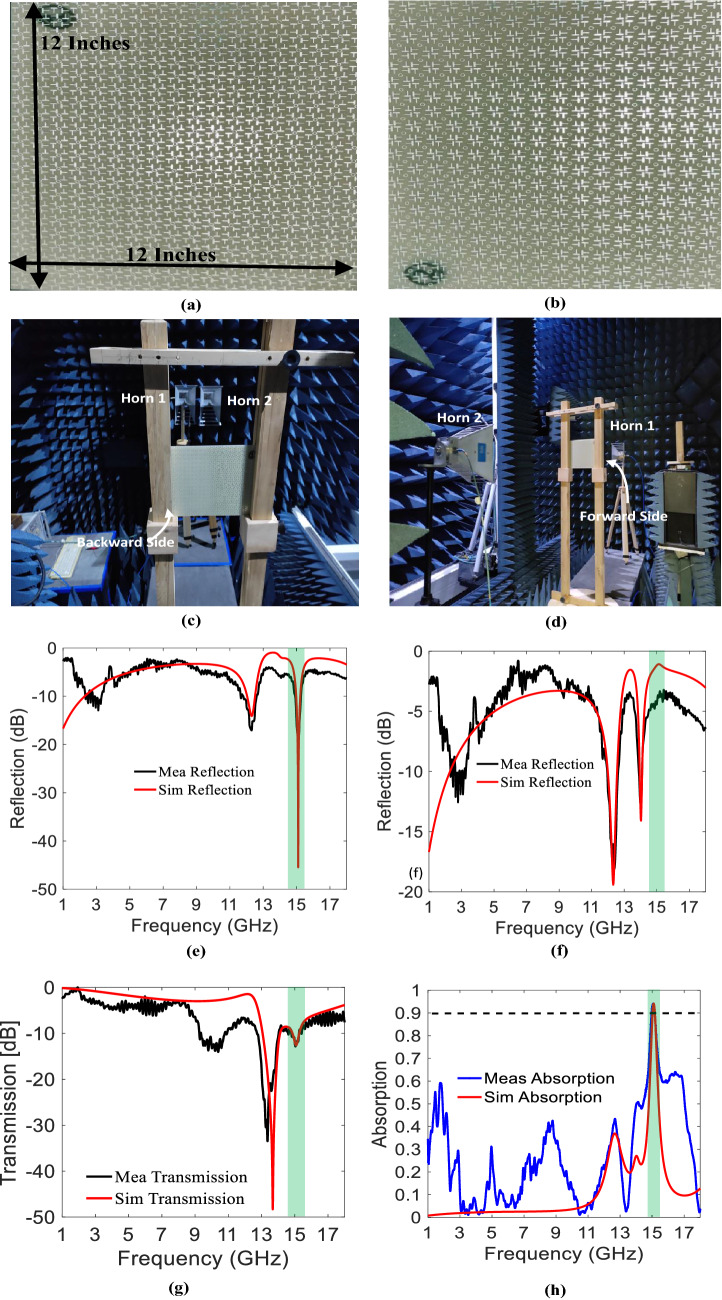
(**a**) Forward illuminating side. (**b**) Backward illuminating side. (**c**) Setup for reflection measurements. (**d**) Setup for transmission measurements. (**e**) Reflection Measurements for forward illuminating side. (**f**) Reflection measurements for backward illuminating. (**g**) Transmission measurements for forward illuminating side. (**h**) Absorption measurements (*The figures (**e–h**) are created using MATLAB ver. R2020A—URL: https://www.mathworks.com/products/matlab.html).

A prototype of the designed metasurface with an area of 300 × 300 mm^2^ is fabricated, depicted in Fig. 7a,b. The fabricated prototype consists of 25 × 25-unit cells, printed on a 2.4 mm thick FR-4 laminate. To authenticate the simulation results, an experimental setup is formed inside an anechoic chamber, shown in Fig. 7c,d. Two wideband horn antennas (1–18 GHz bandwidth) connected to a vector network analyzer (VNA) are utilized for illuminating the metasurface and accepting the reflected/transmitted waves. The cross-reflections and transmissions are not considered in measurements as these were negligible in the simulations. For co-reflection measurements, the horns are positioned at the same side of the metasurface (Fig. 7c), while for co-transmission measurements, they are positioned at the opposing sides (Fig. 7d). Before measuring the actual metasurface, measurements using a solid metal plate and free space were performed as a reference for reflection and transmission, respectively. The measurement results after calibration are plotted in Fig. 7e–h, which show decent agreement with simulations. The variations between simulation and measurement results are due to the finite size of the fabricated prototype and environmental effects. The asymmetric reflection phenomenon due to omega coupling can be clearly seen from Fig. 7e,f.

The comparison with previously published bi-anisotropic surfaces are given in Table 1. It can be seen that the proposed metasurface outperforms the related published work in terms of out-of-band transmission characteristics.

**Table 1:** Comparison with other bianisotropic metasurfaces.

Ref	MA (%)	No of OBT bands	PI	MT
^9^	95	Few MHz	No	16%
^10^	99	Few MHz	Yes (both LP and CP)	7%
^11^	99	Single resonance	Yes (only for LP)	97%
This work	95	L-X band	Yes (both LP and CP)	99% L band, ˃ 50% from S-X band)

*MA* maximum absorption, *OBT* out of band transmission, *PI* polarization insensitive, *MT* maximum transmission, *LP* linearly polarized wave, *CP* circularly polarized wave.

## Conclusion

In this article, a multifunctional bi-anisotropic omega metasurface with ultra-wide out-of-band transmission properties is presented. The proposed omega metasurface absorbs the EM wave at 15.1 GHz with 95% efficiency. The metasurface behavior remains consistent when exposed to oblique incidences, LCP, RCP and any polarization between TE and TM polarizations. The distinct feature of this metasurface is ultra-wide out-of-band transmission ranging from L- to X-band. This metasurface can be used for different applications at a time e.g., high gain and low RCS Fabry Perot Cavity antennas, EMC/I shielding, selective multi-frequency bolometers, ultrathin wave trapping filters, and sensors^21^.

## Data Availability

The datasets generated during and/or analyzed during the current study are available from the corresponding author on reasonable request.
